# High‐Performance Electrochemical Adhesives Enabled by Perfluorinated Sulfonic‐Acid Ionomers with Precise Adhesion Control and Long‐Term Switchability

**DOI:** 10.1002/advs.202510512

**Published:** 2025-08-21

**Authors:** Yoon‐Je Choi, Seungju Lee, Younghoon Kim, Sehwan Park, Hyeong Jun Kim

**Affiliations:** ^1^ Department of Chemical and Biomolecular Engineering Sogang University Seoul 04107 Republic of Korea

**Keywords:** electro‐adhesion, electrochemical adhesion, fracture mechanics, perfluorinated sulfonic‐acid ionomers

## Abstract

Electrically switchable adhesion enables the dynamic and on‐demand modulation of adhesion through electrical control. Herein, a new class of electrochemical adhesives based on the perfluoro‐sulfonic acid (PFSA) ionomers is presented, which deliver robust adhesion with precise tunability through modulation of the applied charge density. Remarkably, once activated, these adhesives maintain strong adhesion without continuous power input, enabling long‐term programmable bonding. Electrochemically tunable adhesion between PFSA ionomers and copper substrates is investigated. A positive potential to the copper substrate promotes sulfonate‐copper coordination at the interface, resulting in strong adhesion, whereas a negative bias triggers detachment. Fracture mechanics analysis reveals a linear relationship between the critical energy release rate (*G_c_*) and applied charge density (*q*) with a value of 3.2  ×  10^−2^ J C^−1^, confirming that electrochemical reactions govern adhesion and can be precisely modulated by the applied charge density. Based on PFSA ionomers, it demonstrates high‐performance electrochemically activated lap‐shear clutches capable of sustaining a force capacity (*F_c_*) of 200 N cm^−2^ with an on/off ratio of 462, maintaining more than 2 months of adhesion retention without a power supply and enduring more than 50 on/off adhesion cycles for repeated use—outperforming existing electrochemical adhesives.

## Graphical abstract

Electrochemical adhesion provides stable, long‐term adhesion without continuous power input after electro‐programming, enabling applications that require durable and controllable adhesion, such as biomedical patches, structural health monitoring sensors, and aerospace devices. Herein, we demonstrate high‐performance electrochemical adhesives based on a perfluoro‐sulfonic acid (PFSA) ionomer coordinated with a copper substrate, enabling precise adhesion control, high on/off adhesion switchability, repeatability, and long‐term adhesion without a continuous voltage supply.

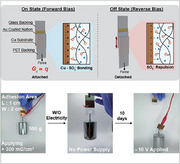

## 1 Introduction

Switchable adhesives dynamically modulate their adhesion properties in response to external stimuli,^[^ ^1^, ^2^ ^]^ including light,^[^ ^3^, ^4^, ^5^, ^6^ ^]^ electric fields,^[^ ^7^, ^8^ ^]^ thermal variations,^[^ ^9^, ^10^, ^11^, ^12^ ^]^ magnetic fields,^[^ ^13^, ^14^, ^15^ ^]^ and chemical reactions.^[^ ^16^, ^17^, ^18^, ^19^ ^]^ This capability unlocks a range of advanced functionalities, such as robotic grippers that precisely handle fragile objects,^[^ ^20^, ^21^, ^22^ ^]^ wearable medical devices that attach and detach without causing skin irritation,^[^ ^16^, ^17^ ^]^ and adjustable consumer products that adapt to user needs.^[^ ^5^, ^10^, ^18^, ^19^ ^]^ Among the various stimuli for switchable adhesion, electrical activation—known as electro‐adhesives—provides distinct advantages, offering precise, rapid, and residue‐free control over adhesion properties, while being readily compatible with electrically operated systems.^[^ ^7^, ^8^ ^]^ Moreover, electrical control can be precisely programmed and regulated through simple electrical circuit systems, facilitating seamless integration across diverse applications in fields such as robotics,^[^ ^20^, ^21^, ^22^, ^23^, ^24^, ^25^, ^26^, ^27^ ^]^ haptics,^[^ ^28^, ^29^, ^30^, ^31^, ^32^, ^33^, ^34^ ^]^ biomedicine,^[^ ^35^, ^36^, ^37^, ^38^ ^]^ and manufacturing.^[^ ^39^ ^]^

The operating mechanisms of electro‐adhesives can be broadly classified into two distinct categories: non‐Faradaic^[^ ^20^, ^21^, ^24^, ^25^, ^28^, ^29^, ^30^, ^31^, ^32^, ^40^, ^41^, ^42^, ^43^ ^]^ and Faradaic^[^ ^22^, ^23^, ^35^, ^36^, ^37^, ^38^, ^44^, ^45^ ^]^ processes. The former relies on electrostatic interactions between charged surfaces, while the latter involves the formation of chemical bonding through electrochemical redox reactions. Although non‐Faradaic electrostatic adhesives enable fast and reversible switching of adhesion, they typically require extremely high voltages of several kV to operate with a constant power supply and retain their adhesion.^[^ ^7^, ^8^ ^]^ These requirements can pose safety risks, increase system complexity, and compatibility issues with other circuit elements, limiting their integration with compact and portable applications. Recently, low‐voltage electrostatic adhesives operating below 10 V have been developed using polyelectrolyte heterojunctions,^[^ ^40^, ^41^, ^42^ ^]^ where molecular‐scale (∼nm) charge separation at the interface enables strong adhesion at low operating voltages.^[^ ^46^, ^47^, ^48^ ^]^ However, these electrostatic adhesives still require a continuous power supply to maintain adhesion, which fundamentally limits their applicability in areas such as biomedical patches, structural health monitoring sensors, and aerospace applications where stable and controllable adhesion is needed over extended periods.^[^ ^1^, ^2^ ^]^

Emerging as a promising strategy to overcome these limitations, electrochemical reaction‐based adhesives offer a fundamentally different approach for long‐term adhesion retention.^[^ ^22^, ^23^, ^35^, ^36^, ^37^, ^38^, ^44^, ^45^ ^]^ These adhesives use electrochemical reactions to modulate adhesion properties, enabling sustained adhesive forces without requiring constant power input. For instance, Xu et al.^[^ ^35^ ^]^ recently demonstrated that metals and graphite electrodes can be electrochemically adhered to soft ionic conductors, such as hydrogels and tissues, using a 5.0 V DC electric field. This electrochemical adhesion can achieve a strength of ≈150 kPa, retaining its adhesive properties without the external electric field for several months. In addition, Bhuiyan et al.^[^ ^45^ ^]^ presented catechol‐based hydrogel electrochemical adhesives activated under a 3.0 V by controlling catechol group exposure and shielding through water electrolysis‐driven reversible cleavage and reformation of the borate ester moiety. These hydrogels achieved an adhesion strength of 11.5 kPa with a rapid switchable adhesive state in under 5 s, further demonstrated in electrically programmable adhesives for climbing robots.^[^ ^23^ ^]^ Although these examples demonstrate the potential of electrochemical adhesives for sustained and reliable adhesion, most current studies often remain at a simple on/off demonstration level, largely due to the limited understanding of the underlying mechanisms and the difficulty of reliably measuring adhesion properties of soft and viscoelastic hydrogels.^[^ ^22^, ^23^, ^35^, ^36^ ^]^ Moreover, the materials used for electrochemical adhesives are largely restricted to soft hydrogels, which suffer from water evaporation, low adhesion performance, cohesive failures, and limited stability, significantly constraining their broader applicability.^[^ ^22^, ^35^ ^]^ Therefore, it is essential to develop stable, high‐adhesion performance electrochemical adhesives with an understanding of the principle for precise control of adhesion strength.

**Figure 1:**
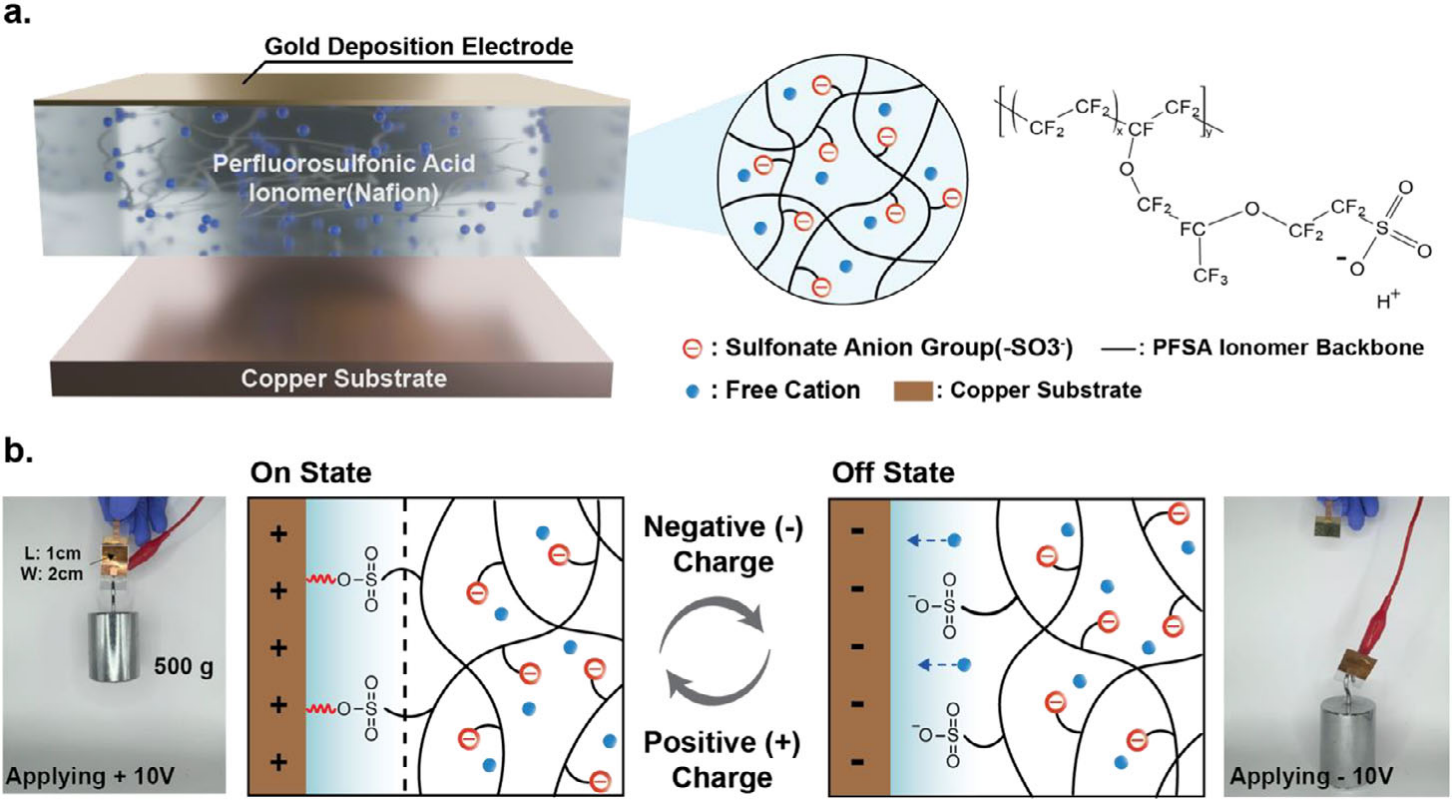
PFSA ionomer‐based electrochemical adhesives. a) Schematic illustration of the electrochemical adhesive system based on PFSA ionomer. b) Mechanism of electrically controlled attachment and detachment at the PFSA ionomer/copper substrate interface.

In this study, we present high‐performance electrochemical adhesives based on a perfluoro‐sulfonic acid (PFSA) ionomer, representing the first demonstration of precise, charge‐regulated control of adhesion through fracture mechanics modeling—advancing well beyond the conventional on/off paradigm of electrochemical adhesives. A thin Au electrode is thermally deposited onto one side of the PFSA ionomer film, forming a parallel‐plate structure with the target metal surface – specifically, a copper substrate – which can reversibly adhere to the PFSA ionomer (**Figure** **1**a). Under positive bias, sulfonate groups in PFSA form a chemical bond with the copper substrate, inducing strong adhesion, while negative bias triggers detachment between PFSA and the copper substrate (Figure 1b). XPS analysis reveals a progressive shift in the O 1s peak from 535.5 eV, corresponding to sulfonate groups (─SO_3_^−^) to 531.8 eV, characteristic of copper‐sulfonate complexes (Cu‐SO_3_ coordination), as the applied charge density increases. The formation of copper‐sulfonate complexes enables the electrochemical attachment, supporting at least 500 g over a 2 cm^2^ adhesion area without an external power source for more than 10 days (Video S1, Supporting Information). Based on the fracture mechanics‐based lap shear joint model, we demonstrate a linear correlation between the critical energy release rate (*G_c_*) under applied charge density (*q*), suggesting that adhesion can be precisely tuned by controlling the applied charge density. This electrochemical adhesive exhibits stable switchability, maintaining stable and repeatable performance over 50 on/off adhesion cycles. The adhesion strength reversibly increases to 6.1 ± 0.80 N cm^−^^2^ under + 6 mC cm^−^^2^ and decreases to 0.7 ± 0.36 N cm^−^^2^ under – 6 mC cm^−^^2^. Furthermore, once activated, the adhesion was maintained for over two months under ambient conditions, exhibiting *F_c_* comparable to that of freshly prepared samples, without the need for continuous power input. By controlling the backing substrate compliance, we achieve a maximum *F_c_* of 200 N cm^−2^ (= 2 MPa), along with an adhesion on/off ratio of 462, which, to the best of our knowledge, surpasses previously reported electrochemical adhesives by an order of magnitude, while maintaining a low operating voltage below 10 V. These findings integrate fundamental principles of molecular design, fracture mechanics, and surface electrochemistry to guide the development of next‐generation electrochemical adhesives.

## 2 Results and Discussions

### 2.1 Fracture Mechanics Analysis of Lap Shear Test on PFSA Ionomer Electrochemical Adhesives

**Figure 2:**
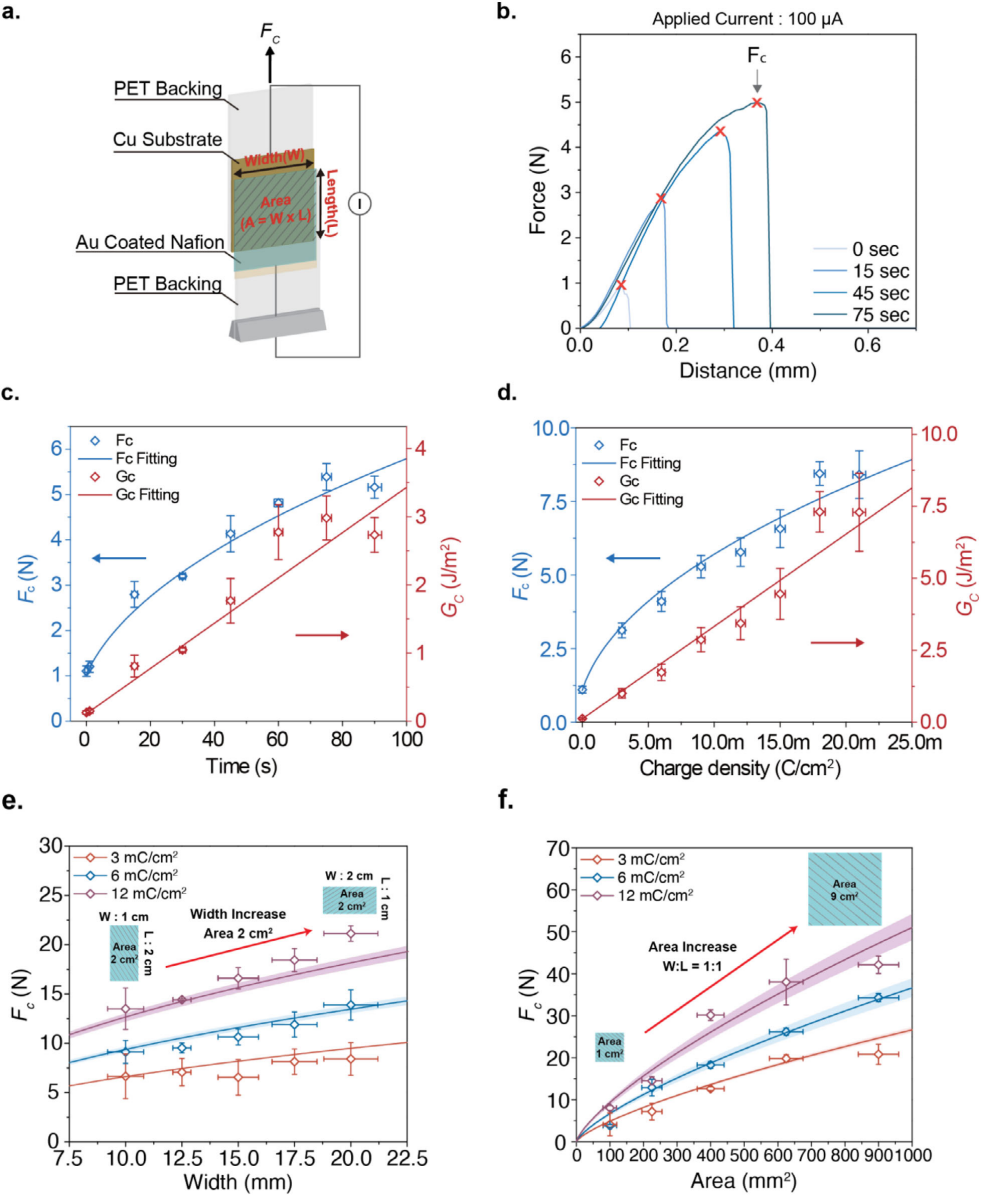
Fracture mechanics analysis of PFSA ionomer‐copper electrochemical adhesive. a) Schematic illustration of the lap shear test structure. b) Force‐displacement curve of the lap shear test under an applied current of 100 µA for a 1 cm^2^ square geometry over increasing time. The red points indicate the *F_c_* of each curve. c) *F_c_* and *G_c_* as a function of time under an applied current density of 100 µA cm^−2^, and d) *F_c_* and *G_c_* as a function of charge density at different levels of applied current density with chargning time of 60 seconds. Blue points indicate the experimental *F_c_* data, and the blue line is a fitting result of the *F_c_* data. e) Lap shear test result of different widths under a uniform area of 2 cm^2^ and f) different areas under a square dimension. Each data point represents the experimental *F_c_* of the lap shear test at various widths and charge densities. The red, blue, and purple line indicates the expected *F_c_* of the lap shear test result under 3, 6, and 12 mC cm^−2,^ with a standard deviation of the shaded regions.

To quantitatively characterize the electrochemical adhesion properties, lap‐shear tests were performed, measuring the *F_c_* at the point of interfacial failure under shear loading. A thin gold electrode (50 nm) was thermally deposited onto one side of the PFSA ionomer surface, enabling the application of an external electrical potential. Both the gold‐coated PFSA ionomer and the copper foil substrate were mounted onto polyethylene terephthalate (PET) backing layers with a thickness of 132 µm, to mechanically prevent stretching or deformation during testing (**Figure** **2**a) According to a simple fracture mechanics model, the *F_c_* measured under unidirectional strain can be described by $F_{c}=\sqrt{2G_{c}}\cdot \sqrt{A/C}$, where critical energy release rate (*G_c_*), representing the adhesion energy of this device, and the ratio of the contact area (*A*) to the total compliance of the device (*C_total_*).^[^ ^21^, ^40^, ^41^, ^49^ ^]^ We assume that electrochemical reactions at the interface primarily influence the thermodynamic component of *G_c_*, denoted as *G_0_*,^[^ ^41^, ^46^ ^]^ while the dissipation factor, which accounts for inelastic processes, remains unaffected.

A constant current of 0.1 mA was applied to a sandwiched assembly of PFSA and copper substrates, with a contact area of 1cm^2^, for durations ranging from 0 to 90 s using a galvanostat (Gamry Reference 620). We applied galvanostatic control over potentiostatic control to achieve precise regulation over the total applied charge density *q*. The corresponding force–displacement curves are shown in Figure 2b. The *F_c_* is defined as the peak force at which mechanical failure occurs between two substrates, and in our lap shear test, all failures occurred at the interface between the PFSA ionomer and the copper substrate. As the charging time increases, *F*_c_ gradually increases. For example, when a 0.1 mA current is applied for 60 s in 1 cm^2^ square geometry (q = 6 mC cm^−2^), *F*_c_ increases from 1.10 N (at 0 s) to 4.81 N (at 60 s). As shown in Figure 2c, the measured *F_c_* exhibits a square‐root dependence on charging time—and consequently on the total *q* under constant current conditions (*F_c_* $\propto \sqrt{q}$). This correlation, which closely agrees with fracture model prediction (*F_c_* $\propto \sqrt{G_{c}}$) in R‐square of 0.98 (Table S2, Supporting Information), supports that the observed enhancement in *G_c_* arises directly from electrochemical reactions (*q*) occurring at the adhesive interface. To quantitatively estimate the value of *G_c_*, we measured the compliance of each constituent component and modeled the total compliance (*C_total_*) using a simplified spring network (Figure S1 and Equation S1, Supporting Information).^[^ ^21^, ^40^, ^41^ ^]^ Then, we calculated the corresponding *G_c_* values using the relation $G_{c}=1/2\;F_{c}^{2}\cdot C_{total}/A$, and plotted them in Figure 2c as a function of charging time. Notably, *G_c_* increases linearly with charging time with linear fitting yielding a slope of 3.3 × 10^−2^ J s m^−2^ under a constant current density of 0.1 mA cm^−2^, indicating that the electrochemical reaction directly governs the increase in *G_c_*_._ The temperature change during the 60 s charging period was less than 1 °C, suggesting that thermal effects are negligible in our system. In addition to varying the charging duration, lap‐shear tests were conducted at different current levels while keeping the charging time fixed at 60 s with a contact area of 1cm^2^ (Figure 2d). Under the identical charging duration, increasing the applied current level led to a corresponding increase in *F_c_*. For example, *F_c_* increases from 4.1 to 8.4 N at the current level of 0.1–0.3 mA, respectively. For a general comparison, the resulting *F_c_* values were plotted as a function of applied *q*, revealing a square‐root dependence (*F_c_* $\propto \sqrt{q}$) with 0.97 of R‐square value from the fitting data (Table S2, Supporting Information; Figure 2d). Similarly, the calculated *G_c_* increases linearly with applied *q*. The linear fitting yields a slope of 3.2 × 10^−2^ J C^−1^, which is closely matched with the result of 3.3 × 10^−2^ J s m^−2^ (equivalent to 3.3 × 10^−2^ J C^−1^) under a constant current density of 0.1 mA cm^−2^. Therefore, these results confirm that *G_c_* can be linearly modulated by the amount of applied charge to the electroadhesive system.

To further validate the fracture mechanics model in predicting the performance of our electrochemical adhesive system, we measured the *F_c_* across varying geometric parameters, including different widths (*W*; Figure 2e) and overlapping areas (*A*; Figure 2f). Notably, under the same overlapping area, *C_total_* decreases with increasing width‐to‐length (*W/L*) ratio (Supporting Information for details). *F_c_* of PFSA ionomer was measured at different applied charge densities, 3, 6, and 12 mC cm^−2^, corresponding to *G_c_* values of 1.07 ± 0.06, 2.02 ± 0.12, and 3.92 ± 0.24 J m^−2^, respectively, under a fixed contact area of 2 cm^2^ while varying overlap width from 1 to 2 cm (Table S2, Supporting Information and Figure 2d). The measured *F_c_* values showed R‐square values of 0.82, 0.92, and 0.96 for 3, 6, and 12 mC cm^−2^, respectively, compared to the predicted value as shown in Figure 2e, Equation S2, and Table S3 (Supporting Information). Increasing the overlap width while maintaining a constant contact area reduces *C_total_*, thereby enhancing *F_c_*. Additionally, we fixed the *W/L* ratio and systematically varied the overlapping area to investigate the geometric dependence of adhesion performance. *F_c_* was measured at the two different charge densities of 3, 6, and 12 mC cm^−2^ as shown in Figure 2f, Equation S3 (Supporting Information). The experimentally measured *F_c_* values showed R‐squares of 0.95, 0.99, and 0.94, respectively, compared to the model‐predicted values derived from the fracture mechanics framework (Table S3, Supporting Information), further validating the robustness of the predictive model. Collectively, these results demonstrate that the *F_c_* of the PFSA ionomer‐based electrochemical adhesives can be precisely modulated by the applied charge density, and its mechanical performance can be reliably predicted using a simplified spring network model‐based fracture mechanics framework. The complete dataset illustrating the effect of system parameters—including charging time, total current, width dependence, adhesion surface area, and copper surface cleaning—is summarized in Figure S2 (Supporting Information).

### 2.2 Surface Analysis of PFSA Ionomer Adhesives Upon Electrochemical Activation

**Figure 3:**
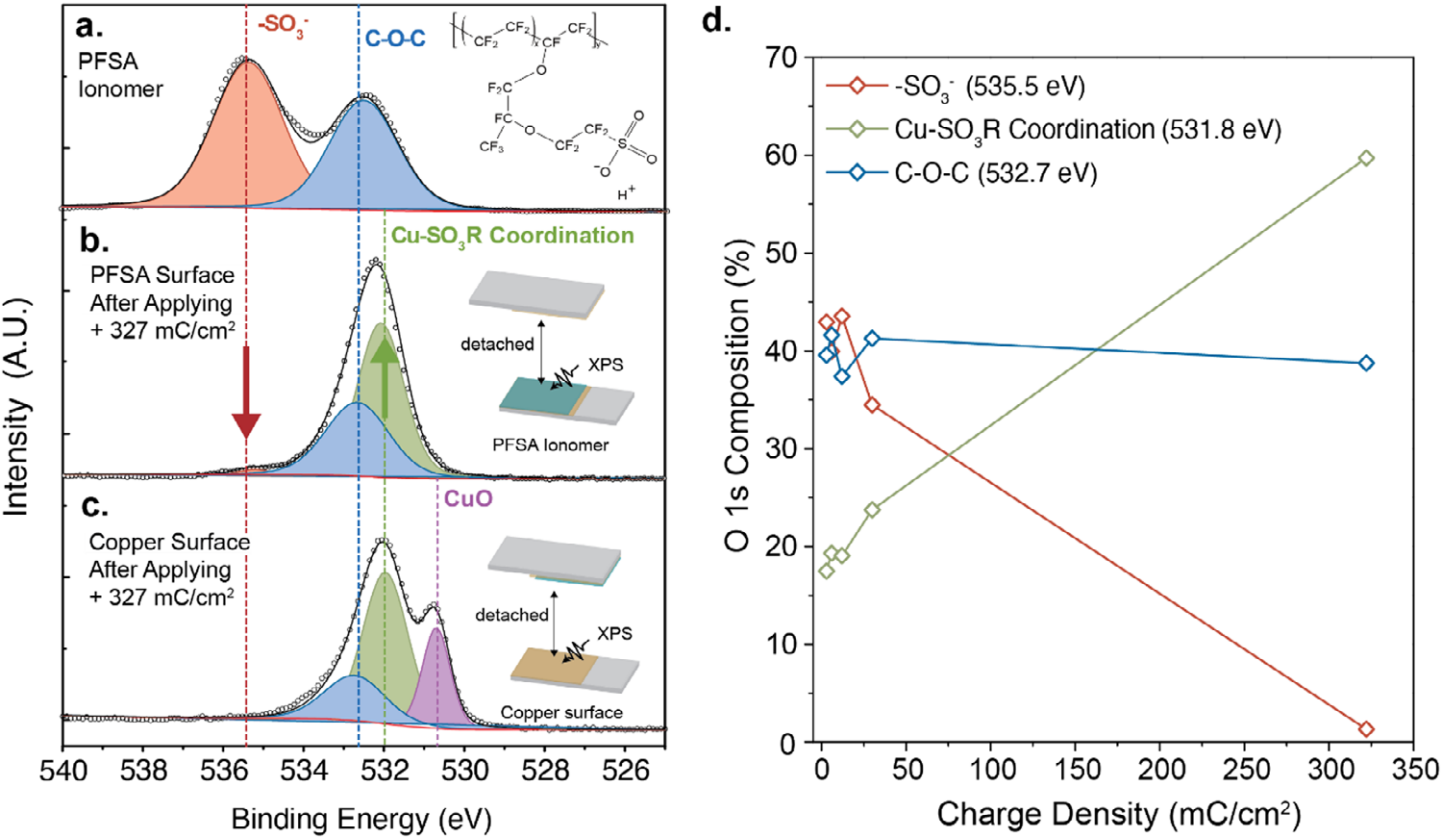
Comparison of O 1s XPS spectra and compositional evolution upon electrochemical activation. a) O 1s XPS spectra of pristine PFSA ionomer, and spectra from mechanically detached surfaces of b) PFSA ionomer film and c) the copper substrate after electrochemical activation at a total charge density of 327 mC cm^−2^. d) Evolution of the O 1s composition in the PFSA ionomer under as a function of applied charge density, showing relative changes in ‐SO_3_‐ (535.5 eV), Cu‐SO_3_R (531.8 eV), and C‐O‐C (532.7 eV).

To investigate the mechanism of electrochemical adhesion formation between the PFSA ionomer and the copper substrate, X‐ray photoelectron spectroscopy (XPS, Kratos Axis Supra ESCAII) was performed. The O 1s binding energies of the ionomer and copper surfaces, before and after electrochemical activation, are shown in **Figure** **3**. The O 1s XPS spectrum of the pristine PFSA ionomer shows two distinct peaks at 535.5 and 532.7 eV, corresponding to the two main oxygen species: sulfonate groups (–SO_3_^−^) and ether linkages (C─O─C) between C─F and CF_2_ units, respectively (Figure 3a). We set a 10 mA current for 10 min to drive extensive electrochemical reactions at the interface, resulting in an actual applied charge of 327 mC cm^−2^ (Figure S3, Supporting Information). Then, the adhered surfaces were mechanically separated, and both surfaces were analyzed using XPS. Notably, on the PFSA side, the 535.5 eV peak—assigned to ─SO_3_^−^ groups—nearly disappeared, leaving only a single dominant peak (Figure 3b). It is worth noting that prior studies show inconsistency in assigning the O 1s XPS peak in PFSA ionomers, with approximately half attributing 535.5 eV to ─SO_3_^−^ oxygen and the other half to C─O─C.^[^ ^50^, ^51^, ^52^, ^53^, ^54^, ^55^ ^]^ Our findings support the assignment of the 535.5 eV peak to ─SO_3_^−^ oxygen, as its intensity decreases upon application of constant current at the PFSA/copper interface, where ─SO_3_^−^ groups are significantly more prone than C─O─C linkages to engage in electrochemical reactions with the copper substrate. In addition, attenuated total reflectance Fourier‐transform infrared spectroscopy (ATR FT‐IR) analysis of the PFSA ionomer surface reveals a decrease in the ─SO_3_H hydrogen stretching band (3000–2750 cm^−1^) and a shift in the O─H bending vibration from 1750 cm^−1^ to 1600–1670 cm^−1^ after electrochemical activation, supporting the conversion of ─SO_3_H after the reaction (Figure S4, Supporting Information).^[^ ^56^ ^]^

After extensive electrochemical reactions, the remaining O 1s peak appears slightly shifted to a lower binding energy of 532.1 eV compared to the characteristic C─O─C ether peak at 532.7 eV (Figure 3b). We attribute this shift to the emergence of a Cu‐SO_3_R coordination peak overlapping with the C─O─C signal. Previous studies have reported O 1s binding energies ≈532.2 eV for Cu─SO_4_ species, supporting the presence of similar copper‐sulfonate coordination.^[^ ^57^ ^]^ Additional evidence for the formation of Cu─SO_3_R coordination is observed on the copper substrate side (Figure 3c), where a further shifted peak at 531.8 eV—clearly distinguishable from the C─O─C bond—appears, likely transferred to the copper surface during mechanical detachment. To more precisely assign the Cu‐SO_3_R coordination peak, we employed a model sulfonate‐containing small molecule. 1‐ethyl‐3‐methylimidazolium trifluoromethanesulfonate ([EMIM][CF_3_SO_3_]), which features a single ‐SO3 functional group without C─O─C moieties, was dissolved in 0.1 m tetrabutylammonium hexafluorophosphate ([TBA][PF_6_]) acetonitrile electrolyte, and a fresh copper substrate was subjected to electrochemical reaction with [EMIM][CF_3_SO_3_] under three electrode electrochemical cell system. Detailed experimental procedures are described in the supporting information. After the electrochemical reaction, a single sharp O 1s peak at 531.8 eV was detected in the XPS analysis, attributed to the formation of Cu‐SO_3_CF_3_, validating the assignment of the Cu‐SO_3_R peak at 531.8 eV (Figure S5a, Supporting Information).

Based on the assigned O 1s binding energies for sulfonate (─SO_3_^−^, 535.5 eV), copper sulfonate (Cu‐SO_3_R, 531.8 eV), and ether groups (C─O─C, 532.7 eV), we systematically monitored the evolution of electrochemical reactions at the PFSA/copper interface under an applied charge densities ranging from 3 to 327 mC cm^−^^2^ (Figure 3d; Figure S6, Supporting Information). Before applying current, the integrated peak areas for ─SO_3_^−^ and C─O─C exhibited a 3:2 ratio, in good agreement with the expected chemical composition of the PFSA ionomer used in this study. As the charging time increased, the intensity of the ─SO_3_^−^ peak gradually decreased, while the C─O─C peak increased in intensity and shifted to lower binding energies, attributed to the emergence of Cu─SO_3_R species overlapping with the C─O─C region. The deconvoluted O 1s peaks, obtained using CasaXPS software with assigned energies for ─SO_3_^−^ (535.5 eV), Cu–SO_3_R (531.8 eV), and C─O─C (532.7 eV), are shown in Figure 3d as a function of applied charge density. Throughout the charging process, the C─O─C portion remained stable at ≈40% of the total oxygen signal, consistent with the expectation that C─O─C linkages were not involved in the electrochemical reaction. Remarkably, the conversion of ─SO_3_^−^to Cu─SO_3_R is approximately a 1:1 ratio, strongly supporting that ─SO_3_^−^ groups are directly transformed into Cu─SO_3_R species upon charge application, providing compelling evidence that this chemical transformation underlies the observed electrochemical adhesion.

### 2.3 High‐Performance PFSA Electrochemical Adhesives with Long‐Term Switchability

**Figure 4:**
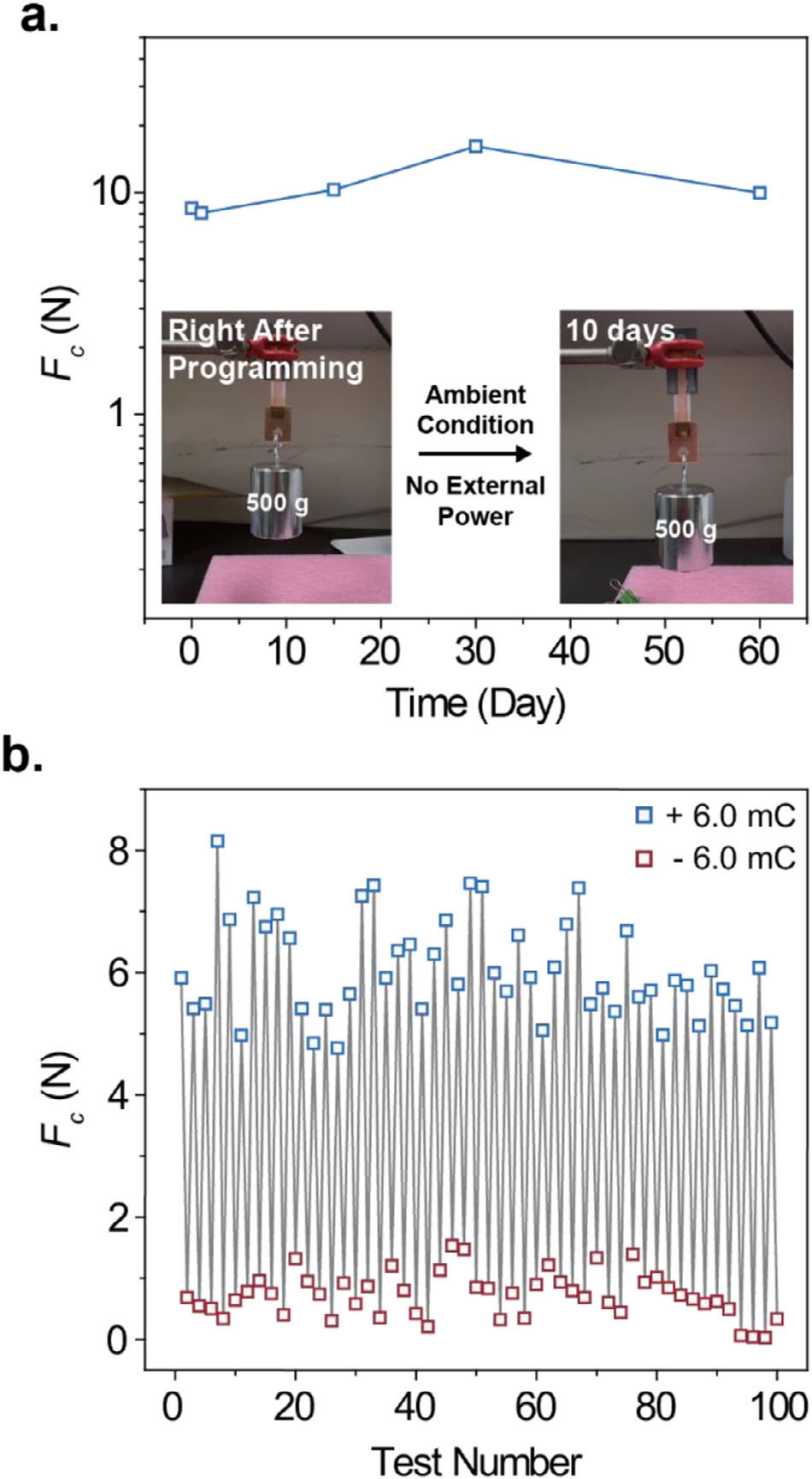
Long‐term switchability and repeatability of PFSA ionomer‐based electrochemical adhesives. a) Long‐term adhesion preservation demonstrated by a lap shear test conducted on a uniform 1 cm^2^ square surface area. The inset shows stable adhesion maintained for over 10 days without any external power input following electrochemical activation. b) 50 on/off adhesion cycles repeatability of the electrochemical adhesive lap shear test result.

We investigate the long‐term switchability and reversibility of PFSA ionomer‐based electrochemical adhesives. A distinctive advantage of these adhesives lies in their ability to retain programmed adhesion states without the need for a continuous power supply, an inherent limitation of non‐Faradaic electrostatic adhesives. To demonstrate this capability, we fabricated a clutch device composed of PFSA ionomer and copper substrates, which were laminated onto a PET backing film. The device featured an overlap area with a 1 cm^2^ square geometry and had an overall width of 1 cm and a length of 5 cm. The device was activated by applying a positive bias current of 10 mA cm^−2^ for 10 min under ambient conditions. Upon activation, the PFSA‐based electrochemical adhesive exhibited an *F_c_* of 10 N cm^−^^2^, corresponding to a shear stress of 100 kPa, sufficient to support a static load of 500 g. Notably, once activated, the adhesion state remained stable without requiring any additional power input. The clutch device retained the applied load for over 10 days under ambient conditions, demonstrating robust long‐term adhesion stability (**Figure** **4**a, inset). To quantitatively assess the durability of the activated state, we systematically measured *F_c_* at various time points after activation, while the device was stored in ambient conditions. The *F_c_* values remained consistent with those observed immediately after activation. For instance, even after ≈2 months of storage under ambient conditions, the device retained a high *F_c_* of 11 N cm^‐^^2^, comparable to that of the freshly activated state. In contrast, applying a reverse (negative) bias switched the adhesives to a detachable state. These results collectively highlight the unique electro‐programmability and long‐term retention capabilities of PFSA‐based electrochemical adhesives, offering a promising platform for applications in soft robotics, reconfigurable electronics, and wearable systems where reversible and persistent adhesion is essential.

The repeatability and reversibility of PFSA‐based electrochemical adhesives are examined through cyclic lap shear test under alternating ± 6 mC cm^−2^ of applied charge conditions over 50 adhesion on/off cycles (Figure 4b). Throughout the cycling process, the adhesion strength remained highly consistent, with a measured *F_c_* of 6.1 ± 0.80 N cm^−^^2^ under + 6 mC cm^−^^2^ and 0.7 ± 0.36 N cm^−^^2^ under − 6 mC cm^−^^2^, confirming reliable on/off switching of adhesion. To directly probe the chemical reversibility underlying this behavior, we performed XPS on the 99th and 100th samples from the 50 cycle test, charged at + 6 mC cm^−^^2^ and − 6 mC cm^−^^2^, respectively (Figure S9, Supporting Information). Both samples showed a constant C─O─C signal at 40%, suggesting no significant degradation of the side chain during the cycling test. Notably, the 99th test sample under + 6 mC cm^−^^2^ exhibited a ─SO_3_^−^ peak at 38% and a Cu─SO_3_R signal at 18%. In contrast, the 100th sample with –6 mC cm^−^^2^ showed a restored ‐SO_3_^−^ peak at 46% and a corresponding decrease in Cu–SO_3_R to 13%, suggesting the reversibility of the coordination reaction between ─SO_3_^−^ and Cu─SO_3_R as the molecular basis of the reversible electroadhesive behavior. To carefully investigate the reversibility of the electrochemical reaction between PFSA and Cu substrate, we employed a bare PFSA ionomer film between the copper electrode and measured cyclic voltammetry (CV) (Figure S10, Supporting Information). By the CV test ranging from −6 to 6 V, the result displays asymmetric and unequal anodic and cathodic peaks, with quasi‐reversible or irreversible redox behavior of the sulfonate group. For more detailed analysis, we conducted CV measurements using a model sulfonate‐containing molecule, [EMIM][CF_3_SO_3_], in a 0.1 m [TBA][PF₆] acetonitrile electrolyte. A clear oxidation peak from the [CF_3_SO_3_]^−^ group was observed at 3.2 V (Figure S8a, Supporting Information), and XPS analysis confirmed the formation of Cu–SO_3_ coordination after this oxidation (Figure S5a, Supporting Information). Similar oxidation peaks were also observed in PFSA solutions within the 3.1–3.7 V range, consistent with the voltage range at which adhesion was activated in lap shear tests (Figure S8b, Supporting Information). While the precise mechanism remains to be elucidated, the consistent switching behavior and XPS analysis suggest that the reversible adhesion of PFSA‐based electrochemical adhesives is governed more by reversible coordination interactions between ─SO_3_^−^ groups and Cu^2^⁺ ions than by classical electron transfer processes. To broaden our PFSA ionomer‐based electrochemical adhesive, lap shear tests are conducted under various conductive substrates (gold, aluminum, indium tin oxide (ITO), and carbon) (see details in Supporting Information). All the substrates showed on/off electrochemical adhesion response under the lap shear test performed after applying ± 6 mC cm^−2^ of charge density (Figure S11, Supporting Information). Except for the copper and gold substrate, other substrates showed a decrease in *F_c_* at the on state (6 mC cm^−2^) as the cycle repeats, suggesting recyclability may depend on substrate‐specific interfacial chemistry.

**Figure 5:**
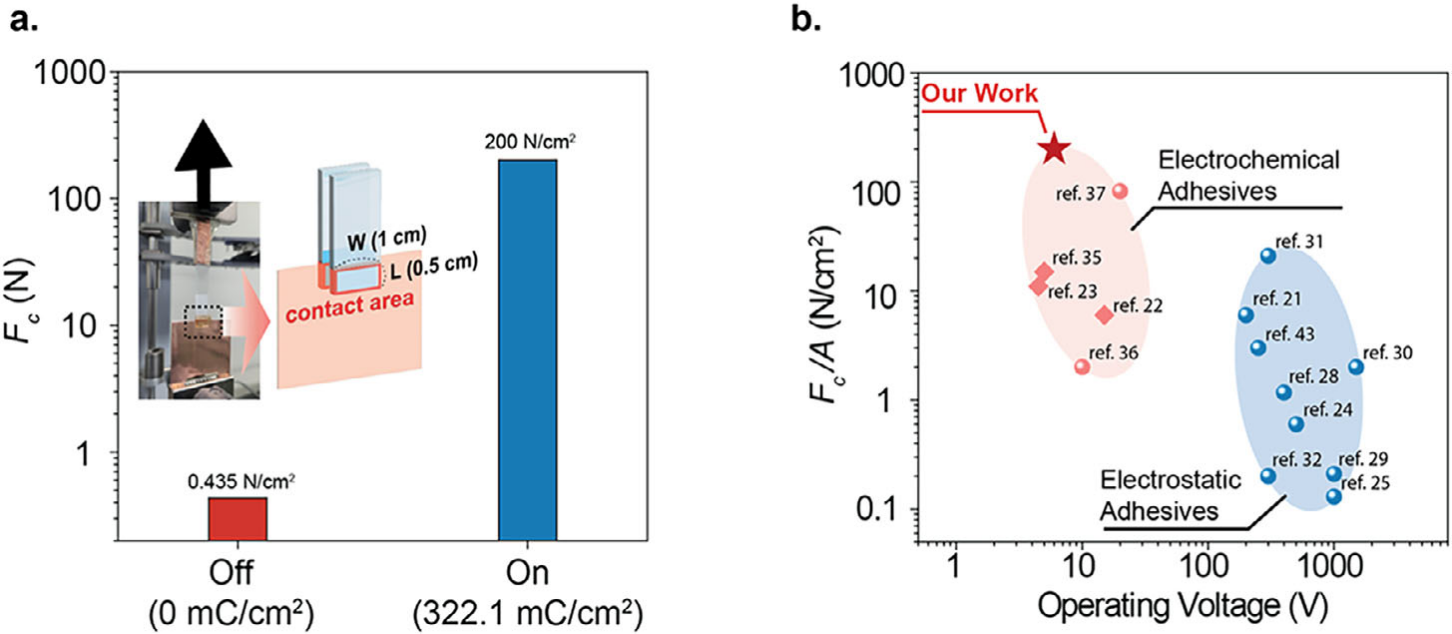
Maximum performance of the PFSA ionomer‐based electrochemical adhesive. a) *F_c_* measurements obtained from tensile testing of the electrochemical adhesive in the “on” state (blue, 322 mC cm^−2^) and off state (red, 0 mC cm^−2^). b) Benchmarking of the best‐performing result from this work against previously reported electrochemical and electrostatic adhesives. Round shape points are the maximum *F_c_* obtained from the shear test, and diamond shape points are the maximum *F_c_* obtained from the normal test.^[^ ^21^, ^22^, ^23^, ^24^, ^25^, ^28^, ^29^, ^30^, ^31^, ^32^, ^35^, ^36^, ^37^, ^43^ ^]^

Lastly, we demonstrate a high‐performance electrochemical adhesion pad based on PFSA ionomers. By reducing the mechanical compliance of the substrate—using glass supports and copper plates with thicknesses of 1.1 and 1 mm, respectively—we can significantly enhance the maximum *F_c_* (**Figure** **5**a). A double lap shear configuration (Figure 5a inset) was employed to minimize bending moments and ensure uniform shear stress distribution across the adhesion interface. Two opposing adhesion areas (1 cm in width and 0.5 cm in length, yielding a total adhesion area of 1 cm^2^) were separated by a 1 mm gap, providing improved mechanical stability over single lap shear tests and effectively isolating the contribution of interfacial adhesion from misalignment. In the “off” state, without applied charge, *F_c_* per unit area was measured to be 0.44 N cm^−2^, corresponding to the friction arising from short‐range van‐der Waals interaction (Figure S12a, Supporting Information). Upon applying a total charge density of 322 mC cm^−2^ in the “on” state (see Figure S12b, Supporting Information for the corresponding current‐time curve), the *F_c_* increased to 200 N cm^−^^2^, yielding an on/off adhesion ratio of 462. In Figure 5b, we compare previously reported best‐performing *F_c_* values per unit area for electrochemical and electrostatic adhesives, plotted against their respective operating voltages. Our adhesives exhibit a best‐performing *F_c_* of 200 N cm^−^^2^, outperforming previously reported electrochemical and electrostatic adhesive systems, which have typically demonstrated values below 20 N cm^−^^2^. This represents more than an order of magnitude improvement in adhesion performance, while maintaining low operating voltages <10 V. The exceptional *F_c_* arises from the synergistic integration of mechanically robust electrochemical adhesives based on PFSA and a device architecture optimized for low compliance, guided by fracture mechanics modeling. This approach represents a significant advancement in the development of low‐voltage, high‐performance electrochemical adhesion systems.

## 3 Conclusion

In this study, we introduce a high‐performance electrochemical adhesive based on PFSA ionomers, featuring precise adhesion control and long‐term switchability. These adhesives can attain an *F_c_* of 200 N cm^−^^2^ with an adhesion on/off ratio of 462, outperforming previously reported electrochemical adhesives. The exceptional performance results from the synergy between the robustness of PFSA ionomers and a low‐compliance device architecture guided by a fracture mechanics model. The electrochemical adhesion mechanism involves electrochemical reaction‐driven interactions between sulfonate groups of the PFSA ionomer and copper substrates, as clearly evidenced by the 1:1 conversion of the SO_3_^−^ to Cu‐SO_3_R peak in XPS analyses. The adhesion strength can be precisely controlled through a demonstrated linear relationship between the *G_c_* and *q* with a correlation of *G_c_* = 3.2 × 10^−2^ J C^−1^, offering a robust methodology for modulating adhesive properties. The long‐term adhesion stability without continuous power input, along with its low‐voltage operation capability, positions it as a promising candidate for applications in soft robotics, wearable medical devices, structural health monitoring sensors, and aerospace technologies, where sustained and reversible adhesion with low‐voltage input is critically important.

## 4 Experimental Section

### Chemicals

Unless otherwise specified, all commercially available chemicals were used as received without further purification.

Acetonitrile (ACN) (HPLC Grade, 99.8%, DAEJUNG Chemicals Co., Ltd.), 1‐ethyl‐3‐methylimidazolium trifluoromethanesulfonate ([EMIM][CF_3_SO_3_]) (Sigma‐Aldrich), tetrabutylammonium hexafluorophosphate ([TBA][PF_6_]) (Sigma‐Aldrich), Nafion dispersion solution 20 wt% (FC international), Nafion 117 (Naracell Tech), gold pellet (Taewon Science), aluminum foil (Wellcos), copper foil (Wellcos), indium tin oxide with polyethylene terephthalate (ITO/PET) (Sigma‐Aldrich), carbon (microporous carbon layer) (Fuelcell Store).

### Fabrication of the Electrochemical Adhesive Film and Substrate Electrode

PFSA ionomer films were sonicated under DI water and soaked overnight. After drying, the PFSA ionomer was transferred into a thermal evaporator, and the gold electrode was deposited at a speed of 0.1–1 nm s^−1^ with a deposition thickness of 50 nm.

### Lap Shear Test

A clutch‐type lap shear samples were prepared with the following geometry for *G_c_* measurement. PET with a thickness of 0.132 mm was used as a backing substrate with a 1 cm width and 4.8 cm length. For uniform compliance, the free length (*L_f_*) of the PET backing has been controlled at 3.5 cm. On the PET backing film, both the PFSA ionomer and the substrate (copper foil, gold‐deposited polyimide film, aluminum foil, and carbon) were mounted using double‐sided tape and sealed with Scotch tape at the end to prevent the substrate from slipping at the backing/substrate interface. The ITO substrate was prepared by directly using an ITO/PET electrode. The overlap region geometry was fixed at 1 cm in width and 1 cm in length, with a 0.146 mm thickness PFSA ionomer (Nafion 117) and a 0.022 mm thickness copper foil. A lap shear test was conducted under unidirectional tension at a test speed of 0.02 mm s^−1^ for all tests using a texture analyzer (Stable Micro Systems, TA.XT Plus).

For the definition of geometrical relationship, the total geometrical contribution was modeled through the simplified spring network model of the lap shear test. For the geometrical dependence definition experiment, all the free length (*L_f_*) of the PET backing was fixed to 1.6 cm. The testing conditions for the geometrical relationship lap shear test are the same as those for the *G_c_* measurement experiment. Each of the models for *F_c_* on width and area difference follows the model equation written in the supporting information. In all experiments, we consistently observed adhesive failure at the exposed PFSA side, not at the PET (or glass)/Au or Au/PFSA interfaces.

### Electrochemical Analysis

All electrochemical analyses were conducted using a potentiostat/galvanostat EIS (Gamry, reference 620). A 3‐electrode electrochemical cell test of [EMIM][CF_3_SO_3_] solutions (20, 40, 80 µL) and PFSA ionomer solutions (20, 40 µL/ 10 mL) was conducted in a 0.1 m [TBA][PF_6_] acetonitrile solution. Glassy carbon was selected as the working electrode, Ag/Ag^+^ in 0.1 m [TBA][PF_6_] acetonitrile solution as the reference electrode, and Pt mesh as the counter electrode for the CV test of the electrochemical cell. With the scan rate of 100 mV s^−1^, we measured five cycles of CV and used the second step as our experimental data.

For XPS analysis, we used copper foil as the working electrode in a three‐electrode electrochemical cell. Chronoamperometry was performed at 3.2 V for 60 s with [EMIM][CF_3_SO_3_] reactant, and 3.35 V for 60 s with the PFSA ionomer solution. For PFSA ionomer film, a gold‐deposited PFSA ionomer was brought into mechanical contact with a copper substrate in a 1 cm^2^ square geometry, and two‐electrode electrochemical measurements were subsequently conducted. Under this geometry, we conduct a CV test with a scan rate of 10 mV s^−1^ and a voltage range of −6 to 6 V. A Copper substrate is used as the working electrode, and a gold layer is used as the counter electrode.

### Surface Optical Analysis

PFSA ionomer film and copper substrate were analyzed on Kratos Axis Supra ESCAII installed at the National Center for Inter‐university Research Facilities (NCIRF) at Seoul National University. The data was processed and fitted using the CasaXPS program. Surface ATR FT‐IR analysis was held using Jasco FT/IR‐6X.

## Conflict of Interest

The authors declare no conflict of interest.

## Supporting information



Supporting Information

Supporting Information 2

Supplemental Video 1

## Data Availability

The data that support the findings of this study are available from the corresponding author upon reasonable request.
