# Supplementary material for: High‐Performance Electrochemical Adhesives Enabled by Perfluorinated Sulfonic‐Acid Ionomers with Precise Adhesion Control and Long‐Term Switchability
Source: Adv Sci (Weinh). 2025 Aug 21;12(42):e10512. doi: 10.1002/advs.202510512 (PMC12622435; doi:10.1002/advs.202510512)
Supplement: Supplementary file 1 — Supporting Information [file ADVS-12-e10512-s002.docx]

Supporting Information

High-Force Capacity Electrochemical Adhesives Enabled by Perfluorinated Sulfonic-Acid Ionomers with Precise Adhesion Control and Long-Term Switchability

Yoon-Je Choi, Seungju Lee, Younghoon Kim, Sehwan Park, and Hyeong Jun Kim*

**Table of Contents**

**Supplementary Text**

1. **Total compliance calculation based on fracture mechanics**
2. **Electrochemical analysis of copper-sulfonate coordination**
3. **PFSA ionomer electrochemical adhesive substrate universality test sample preparation**

**Supplementary Figures & Table**

**Figure S1.** Total compliance calculation design scheme of the PFSA ionomer electrochemical adhesive lap shear joint model.

**Figure S2.** Structural property – charging time, total current, width, surface area, copper substrate cleaning – relationship of PFSA ionomer electrochemical adhesive

**Figure S3.** Chronopotentiometry result of a 1cm^2^ square PFSA ionomer-Copper interface for XPS analysis.

**Figure S4.** ATR FT-IR result of PFSA ionomer surface before and after electrochemical reaction.

**Figure S5.** O 1s XPS result of copper substrate after chronoamperometry test in the electrochemical cell.

**Figure S6.** XPS result of PFSA ionomer under 100 μA/cm2 with different reaction times.

**Figure S7.** Electrochemical cell setup for electrochemical mechanism analysis.

**Figure S8.** Cyclic voltammetry result of the electrochemical cell.

**Figure S9.** XPS surface analysis of PFSA ionomer surface after repeatability test.

**Figure S10.** Cyclic Voltammetry result of PFSA ionomer film in a scan rate of 10 mV/s with copper as the working electrode and gold as the counter/reference electrode.

**Figure S11.** Electrochemical adhesion behavior of PFSA ionomer on different conductive substrates in the "on" state (6 mC/cm²) and "off" state (– 6 mC/cm²).

**Figure S12.** Best performance result of PFSA ionomer electrochemical adhesive

**Table S1.** Property of each material substance used for the *C_total_* calculation of the lap shear test

**Table S2.** *G_c_* Fitting of the electrochemical adhesive lap shear test result

**Table S3.** R-Square of electrochemical adhesive lap shear test result under geometrical dependence

**Table S4.** XPS surface analysis result of the PFSA ionomer electrochemical adhesive surface. Property of each material substance used for the *C_total_* calculation of the lap shear test.

**Supplementary Videos**

**Video S1.** Long-term adhesion endurance of PFSA ionomer electrochemical adhesive

**1. Total compliance calculation based on fracture mechanics**

The Lap Shear clutch model was prepared with the following geometry for adhesion energy measurement. PET with a thickness of 0.132 mm was used as a backing substrate with a 1 cm width and 4.8 cm length. For uniform compliance, the free length (*L_f_*) of the PET backing has been controlled at 3.5 cm. By measuring each material’s Young’s modulus and estimated Poisson’s ratio of PFSA ionomer and copper foil, we could calculate total compliance (*C_total_*) of the material with the following equation achieving 2.046 x 10^-2^ mm/N, dominately governed by the *E_PET backing_* and *L_f, PET backing_* values. (**Equation S1**)

$\boldsymbol{C}_{\boldsymbol{total}}\boldsymbol{=}\frac{\boldsymbol{L}}{\boldsymbol{W}}\left( \frac{\boldsymbol{1}}{\boldsymbol{2}\boldsymbol{E}_{\boldsymbol{PET} \boldsymbol{backing}}\boldsymbol{t}_{\boldsymbol{PET} \boldsymbol{backing}}\boldsymbol{+}\boldsymbol{E}_{\boldsymbol{PFSA} \boldsymbol{ionomer}}\boldsymbol{t}_{\boldsymbol{PFSA} \boldsymbol{ionomer}}\boldsymbol{+}\boldsymbol{E}_{\boldsymbol{Cu}}\boldsymbol{t}_{\boldsymbol{Cu}}} \right)$

$\boldsymbol{+}\frac{\boldsymbol{1}}{\boldsymbol{L*W}}\left( \boldsymbol{t}_{\boldsymbol{PFSA ionomer}}\left( \boldsymbol{1+}\boldsymbol{\upsilon}_{\boldsymbol{PFSA ionomer}} \right)\boldsymbol{+}\boldsymbol{t}_{\boldsymbol{Cu}}\left( \boldsymbol{1+}\boldsymbol{\upsilon}_{\boldsymbol{Cu}} \right) \right)\left( \frac{\boldsymbol{1}}{\boldsymbol{E}_{\boldsymbol{PFSA ionomer}}}\boldsymbol{+}\frac{\boldsymbol{1}}{\boldsymbol{E}_{\boldsymbol{Cu}}} \right)$

$\boldsymbol{+2}\left( \frac{\boldsymbol{L}_{\boldsymbol{f, PET backing}}}{\boldsymbol{E}_{\boldsymbol{PET backing}}\boldsymbol{t}_{\boldsymbol{PET backing}}\boldsymbol{W}} \right)$ (**Equation S1**)

From the calculated result, we elucidate the critical energy release rate (*G_c_*) of the device, which is analogous to the adhesion energy. A lap shear test was conducted under unidirectional tension at a test speed of 0.02 mm/s for all tests, ensuring the *L_f_* of the sample.

For the definition of geometrical relationship, the total geometrical contribution was modeled through the fracture model of the lap shear test. (**Equation 1**) For the experiment, the free length (*L_f_*) of the PET backing was controlled at 1.6 cm. Each of the models for force capacity (*F_c_*) on width (*W*) (**Equation S2**) and area (*A*) (**Equation S3**) difference follows the model equation written below, applying the model to the fitting result. A lap shear test was conducted under unidirectional tension at a test speed of 0.02 mm/s for all tests, ensuring the *L_f_* of the sample.

$\boldsymbol{F}_{\boldsymbol{c}}\boldsymbol{=}\sqrt{\boldsymbol{2}\boldsymbol{G}_{\boldsymbol{c}}}\sqrt{\frac{\boldsymbol{2}}{\boldsymbol{8}\boldsymbol{.}\boldsymbol{892}\boldsymbol{\times}\boldsymbol{10}^{\boldsymbol{-}\boldsymbol{5}}\boldsymbol{+}\boldsymbol{9}\boldsymbol{.}\boldsymbol{218}\boldsymbol{\times}\boldsymbol{10}^{\boldsymbol{-}\boldsymbol{1}}\boldsymbol{/}\boldsymbol{W}\boldsymbol{+}\boldsymbol{5}\boldsymbol{.}\boldsymbol{597}\boldsymbol{\times}\boldsymbol{10}^{\boldsymbol{-}\boldsymbol{1}}\boldsymbol{/}\boldsymbol{W}^{\boldsymbol{2}}}}$ (**Equation S2**)

$\boldsymbol{F}_{\boldsymbol{c}}\boldsymbol{=}\sqrt{\boldsymbol{2}\boldsymbol{G}_{\boldsymbol{c}}}\sqrt{\frac{\boldsymbol{A}}{\boldsymbol{2}\boldsymbol{.}\boldsymbol{799}\boldsymbol{\times}\boldsymbol{10}^{\boldsymbol{-}\boldsymbol{4}}\boldsymbol{+}\boldsymbol{9}\boldsymbol{.}\boldsymbol{218}\boldsymbol{\times}\boldsymbol{10}^{\boldsymbol{-}\boldsymbol{2}}\boldsymbol{/}\sqrt{\boldsymbol{A}}\boldsymbol{+}\boldsymbol{1}\boldsymbol{.}\boldsymbol{778}\boldsymbol{\times}\boldsymbol{10}^{\boldsymbol{-}\boldsymbol{3}}\boldsymbol{/}\boldsymbol{A}}}$ (**Equation S3**)

**2. Electrochemical analysis of copper-sulfonate coordination**

To further solidify our understanding of the electrochemical adhesion mechanism, we performed XPS analysis on a copper substrate reacted under a three-electrode electrochemical cell model system. For the model system, we select the sulfonate-containing small molecule, 1-ethyl-3-methylimidazolium trifluoromethanesulfonate ([EMIM][CF_3_SO_3_]), and a PFSA ionomer solution as reactant materials dissolved in a 0.1 M tetrabutylammonium hexafluorophosphate ([TBA][PF_6_]) acetonitrile electrolyte (**Figure S8a**). We define the electrochemical reaction potential of the sulfonate group through cyclic voltammetry (CV) using a glassy carbon electrode as the working electrode. Glassy carbon is used to prevent working electrode ionization, which hinders the exact definition of the reaction potential. By the CV test ranging from – 5 V to 5 V with a scan rate of 100 mV/s, the electrochemical window of the [TBA][PF_6_] 0.1 M electrolyte is defined to be - 3.1 V– 3.7 V (**Figure S7b**). Next, we add different amounts (20, 40 μl) of [EMIM][CF_3_SO_3_] and PFSA ionomer solution to the electrolyte, conducting CV in the same voltage range of - 5V – 5V. The CV curves displayed new asymmetric and unequal anodic and cathodic peaks, with broad and shifted potentials inside the electrochemical window of the electrolyte, indicating quasi-reversible or irreversible redox behavior of the sulfonate group (**Figure S8**). The increased height of the peaks with an increased amount of added reactant also supports the idea that the peaks originate from the sulfonate group of the reactants. Based on the peak analysis, [EMIM][CF_3_SO_3_] showed 3.2 V of reaction potential, and PFSA ionomer solution showed 3.0 – 3.7 V of broader reaction potential from the CV result. (**Figure S8**)

With the elucidated reaction potential of the sulfonate group, we further conducted copper substrate surface XPS analysis after 60 seconds of chronoamperometry test under reaction potential (Figure S7). For [EMIM][CF_3_SO_3_] small molecule O 1s XPS analysis, one sharp oxygen peak at 531.8 eV is detected, which is 1.3 eV bigger than the CuO peak, indicating that the sulfonate group of [CF_3_SO_3_] anion is attached to the copper substrate, showing Cu-SO_3_R coordination (**Figure S5a**). Additionally, for the PFSA ionomer solution small molecule O 1s XPS analysis, three oxygen peaks corresponding to -SO_3_-, Cu-SO_3_R coordination, and C-O-C are detected (**Figure S5b**). The results show excellent correlation with the O 1s binding energy of the PFSA ionomer electrochemical adhesive, proving that the electrochemical reaction between copper and the sulfonate group governs our adhesion mechanism.

**3. PFSA ionomer electrochemical adhesive substrate universality test sample preparation**

To further expand the substrate universality of our PFSA ionomer electrochemical adhesive, we performed lap shear tests with different conductive substrates (gold, aluminum, indium tin oxide (ITO), and carbon). A gold electrode was prepared using thermal evaporation of a gold pellet onto a polyimide substrate with a thickness of 125 μm at a speed of 0.1-1 nm/s. Aluminum foil was used as an aluminum substrate. ITO was prepared by directly using an ITO/PET electrode. A carbon substrate was prepared using a microporous carbon electrode. Each sample, except ITO, was mounted onto a PET backing substrate with a thickness of 135 μm, a width of 1 cm, and a length of 4.8 cm using double-sided tape. The test sample was sealed using Scotch Tape to prevent slipping between the PET backing and the substrate.

**Supplementary Figures**


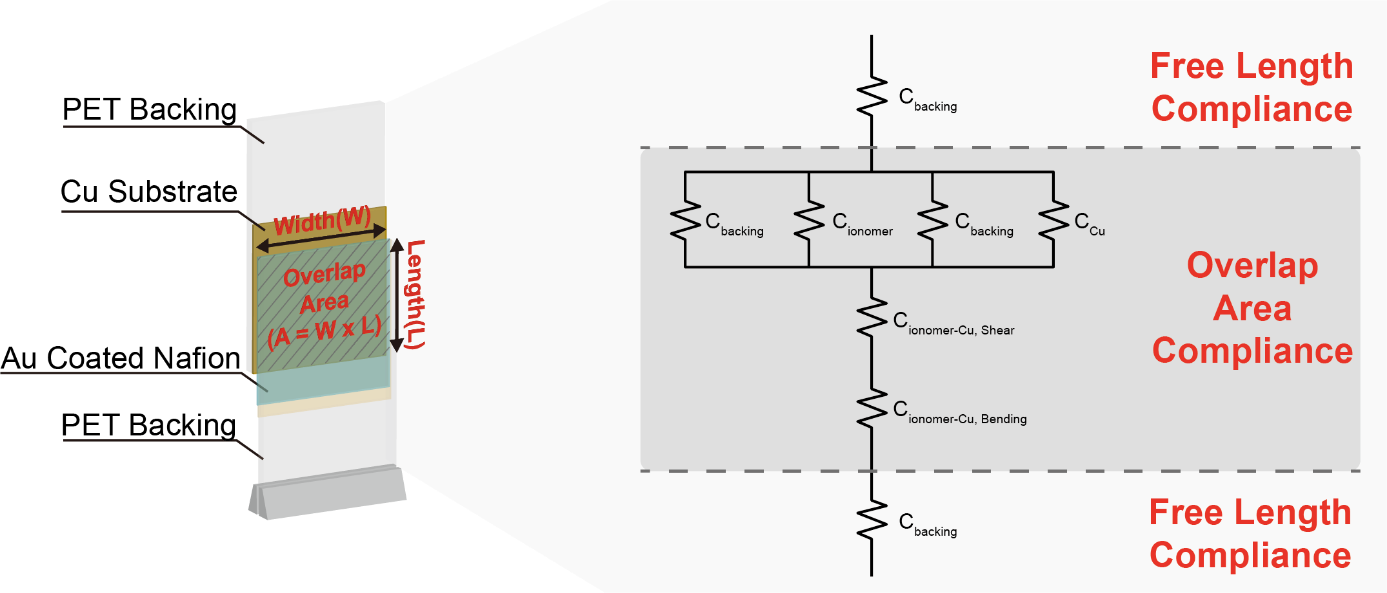


Figure S1. Total compliance calculation design scheme of the PFSA ionomer electrochemical adhesive lap shear joint model.


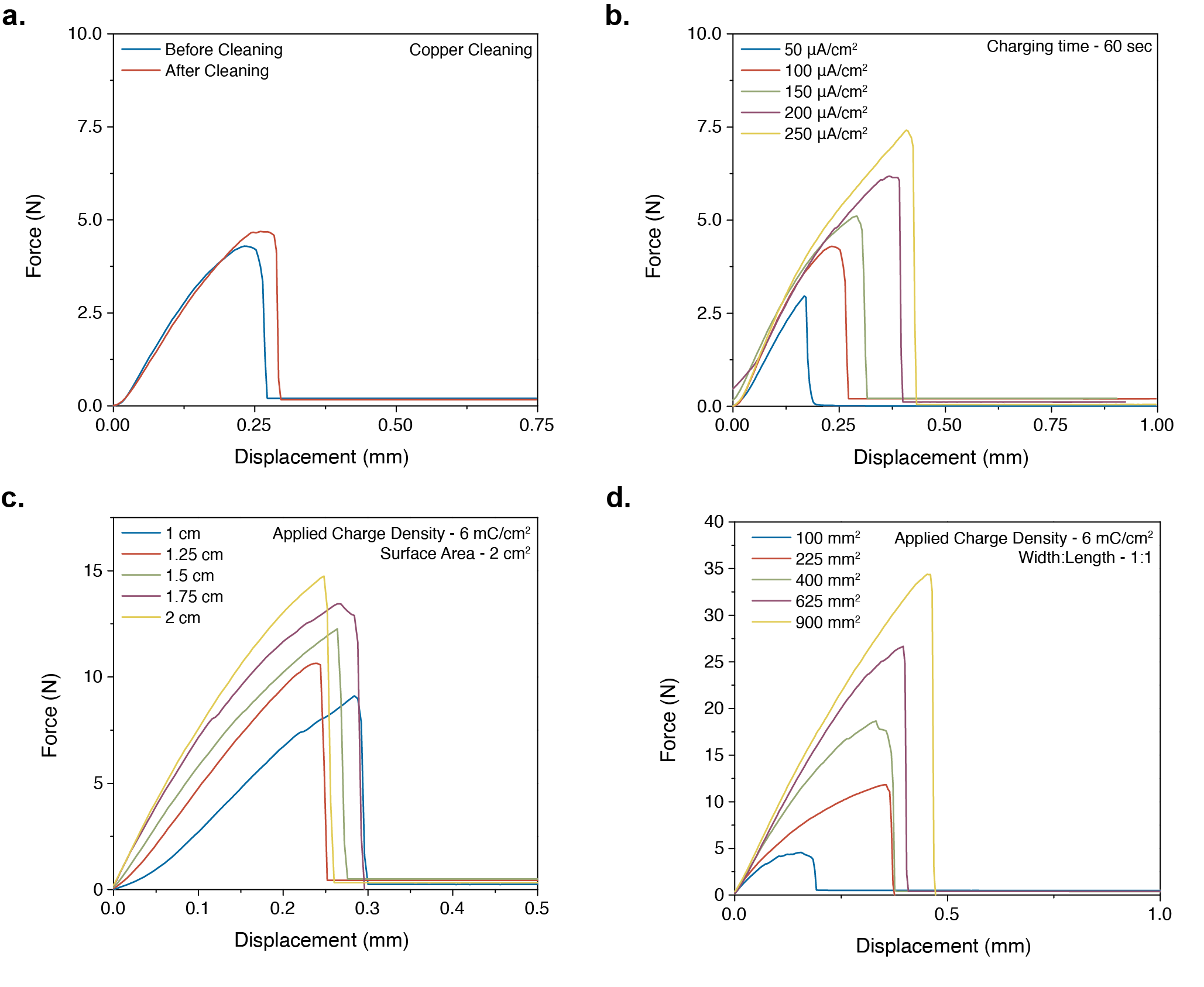


**Figure S2. Structural property – copper substrate cleaning (a.), total current (b.), width (c.), surface area (d.), copper substrate cleaning – relationship of PFSA ionomer electrochemical adhesive.**


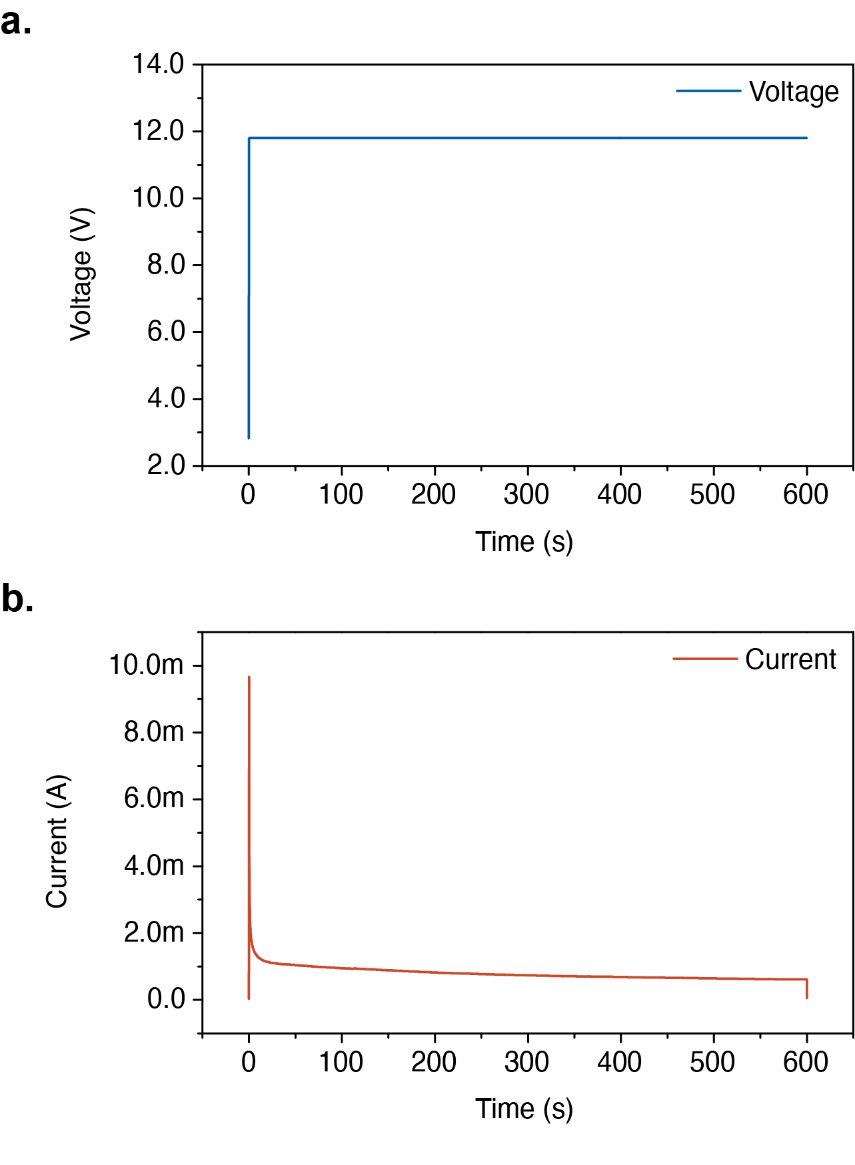


**Figure S3. Chronopotentiometry result of 1cm^2^ square PFSA ionomer-Copper interface for XPS analysis, a.** applied voltage of chronopotentiometry under 10 mA/cm^2^ of maximum current load, and **b.** actual applied current under 10 mA/cm^2^ of maximum current load. The applied *q* of the sample is calculated through the integration of the current-time graph.


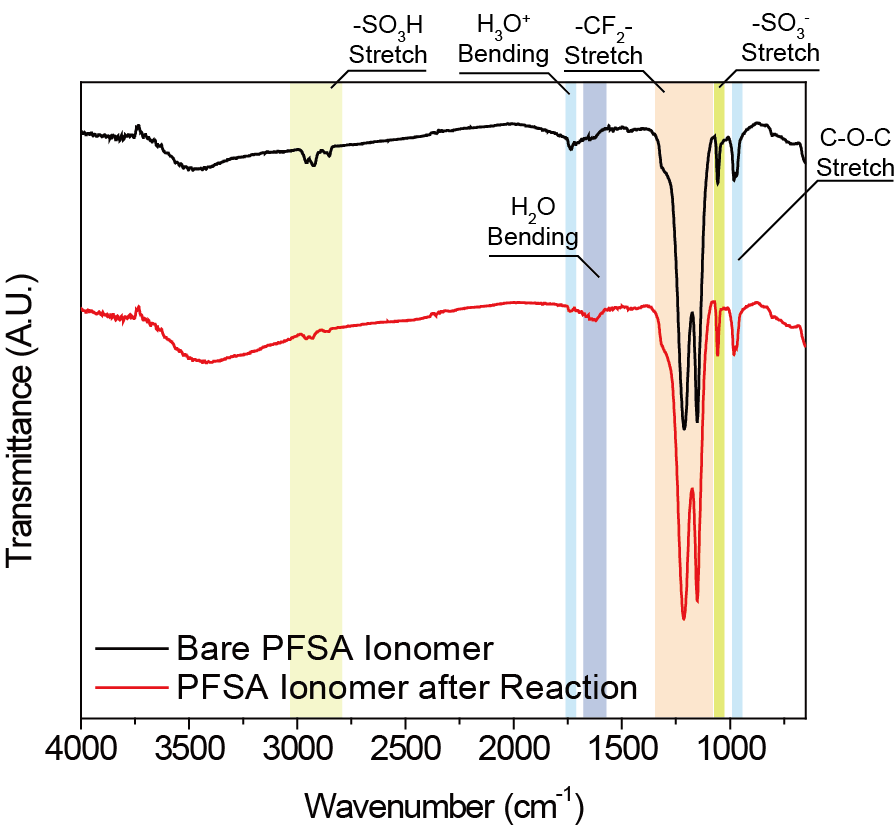


**Figure S4. ATR FT-IR result of PFSA ionomer surface before and after electrochemical reaction.**


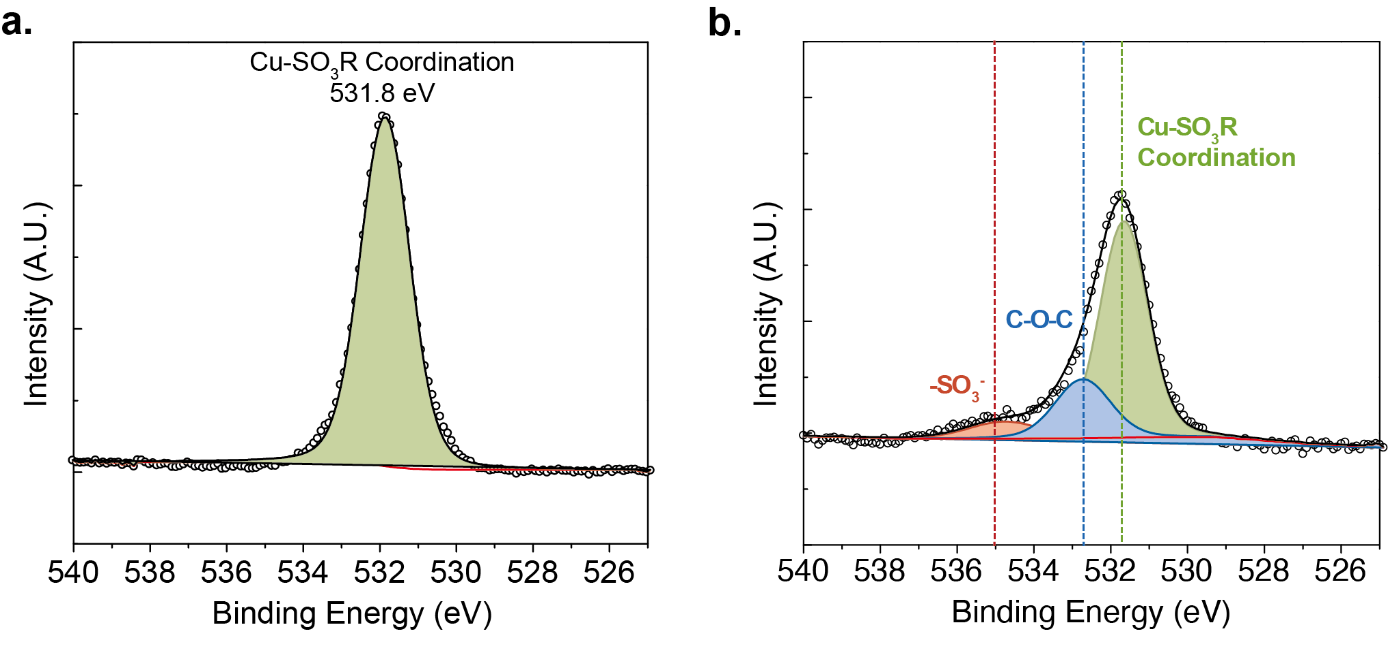


**Figure S5. O 1s XPS result of copper substrate after chronoamperometry test at electrochemical cell a.** with [EMIM][CF_3_SO_3_] as reactant, and **b.** with PFSA ionomer solution as reactant.


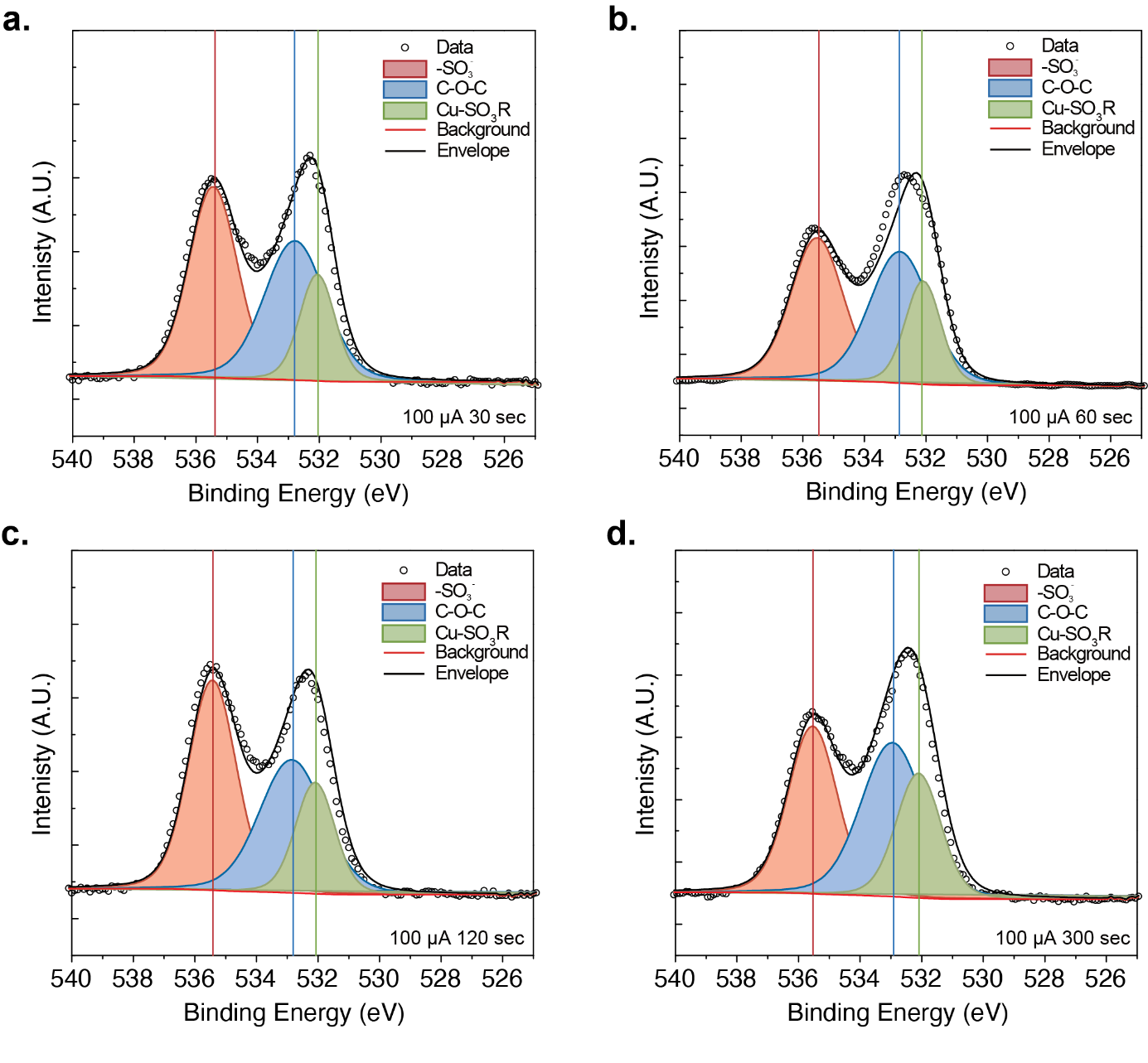


**Figure S6.** **XPS result of PFSA ionomer under 100 μA/cm^2^ with different reaction times.** **a.** After applying 100 μA/cm^2 for^ 30 seconds. **b.** After applying 100 μA/cm^2 for^ 60 seconds. **c.** After applying 100 μA/cm^2 for^ 120 seconds. **d.** After applying 100 μA/cm^2 for^ 300 seconds.


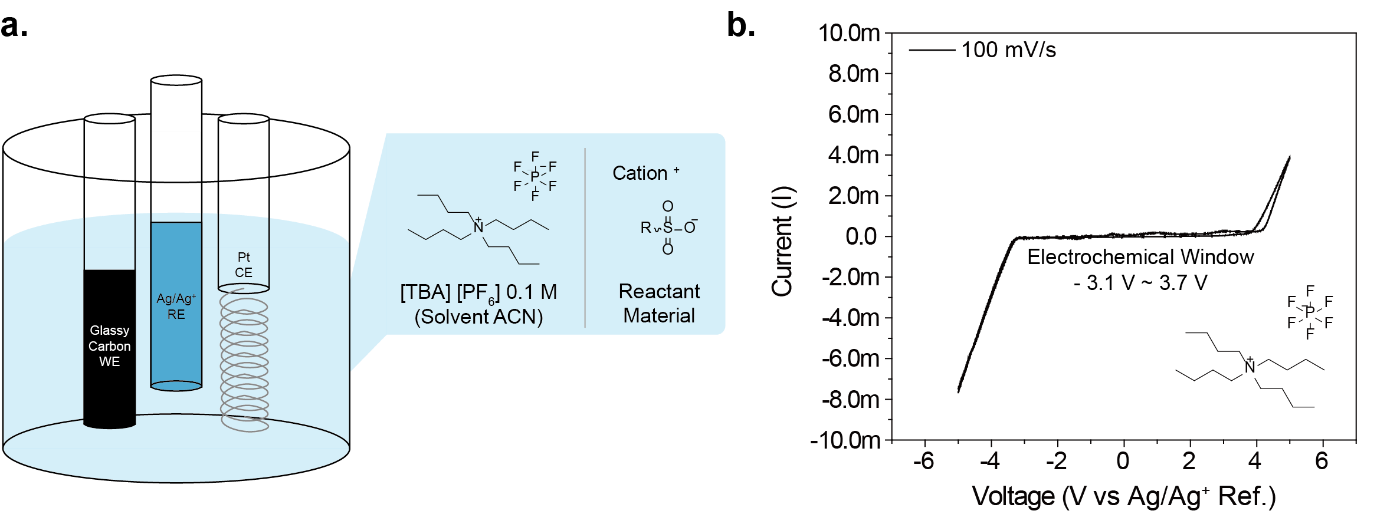


**Figure S7. Electrochemical cell setup for electrochemical mechanism analysis.** **a.** Electrochemical cell structure for cyclic voltammetry test. **b.** Cyclic voltammetry result of [TBA][PF_6_] 0.1 M Acetonitrile electrolyte solution.

**
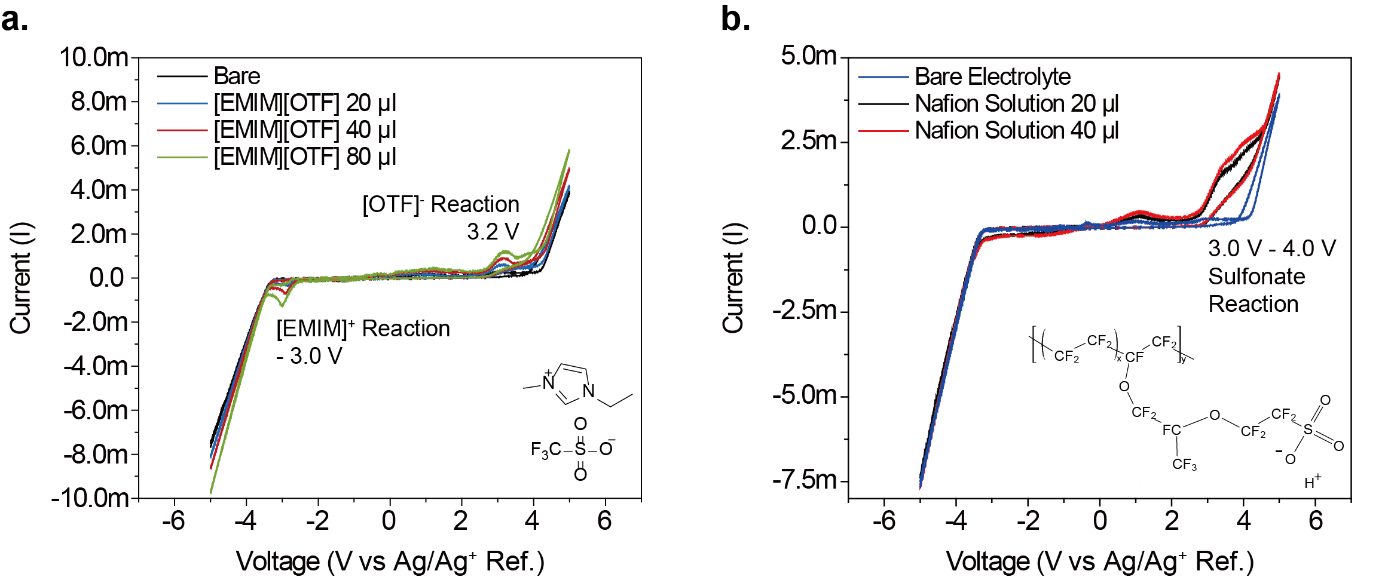
**

**Figure S8. Cyclic voltammetry result of the electrochemical cell.** **a.** [EMIM][OTF] as a reactant molecule with different reactant content. **b.** PFSA ionomer solution as a reactant molecule with different reactant content


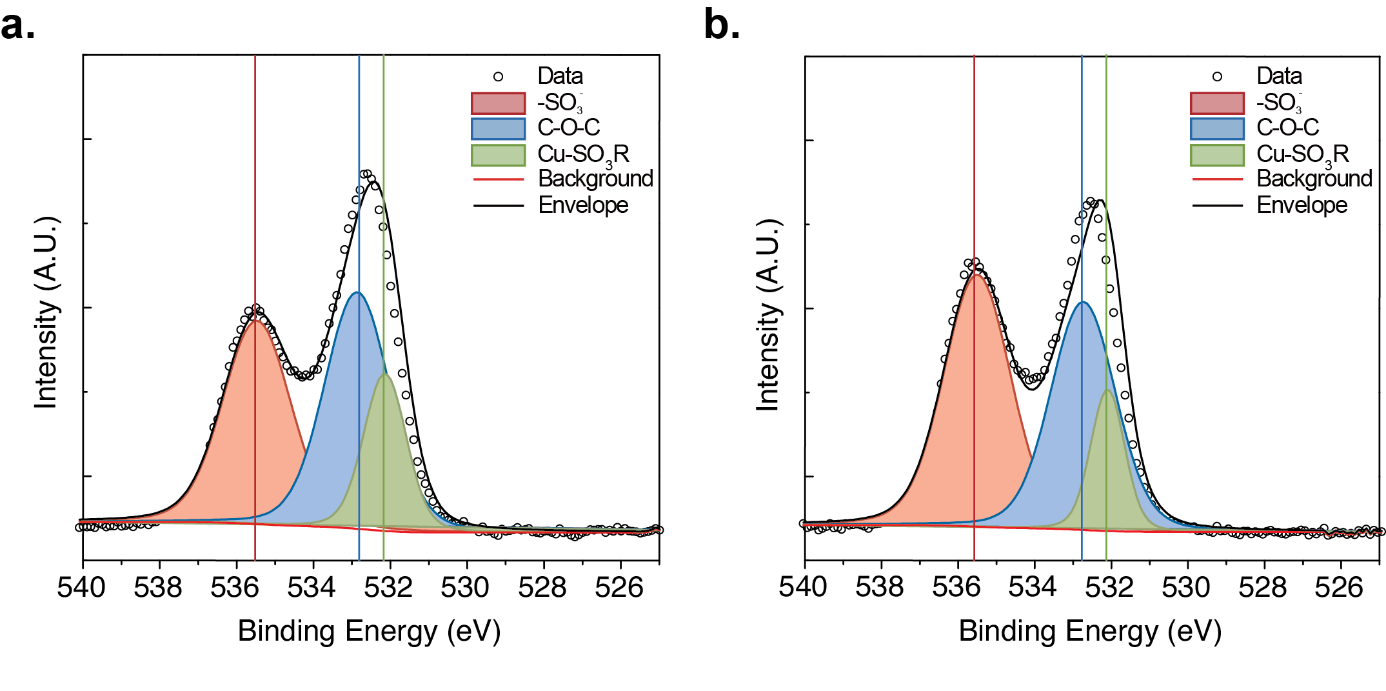


**Figure S9. XPS surface analysis of PFSA ionomer surface after repeatability test. a.** XPS surface analysis of PFSA ionomer surface under the 99^th^ cycle of repeatability test. **b.** XPS surface analysis of PFSA ionomer surface under the 100^th^ cycle of the repeatability test.


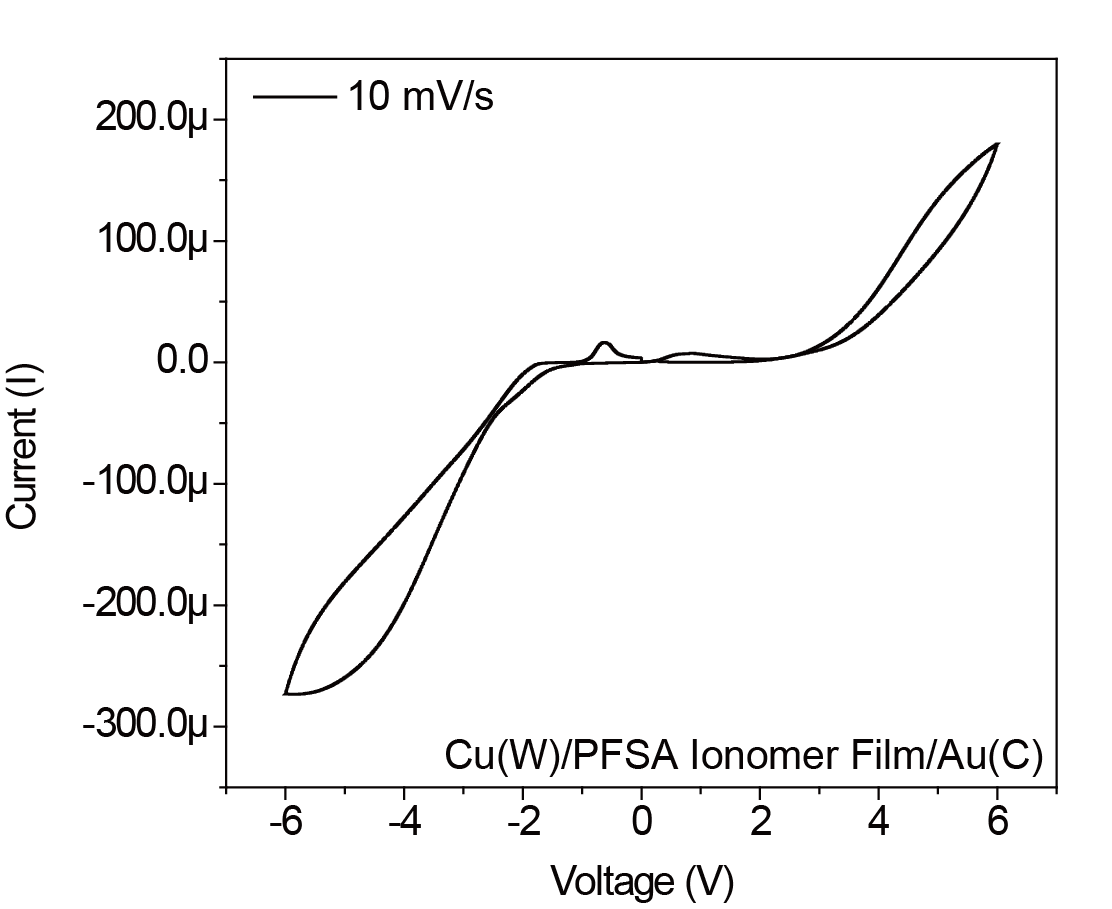


**Figure S10. Cyclic Voltammetry result of PFSA ionomer film in a scan rate of 10 mV/s with copper as the working electrode and gold as the counter/reference electrode.**

**
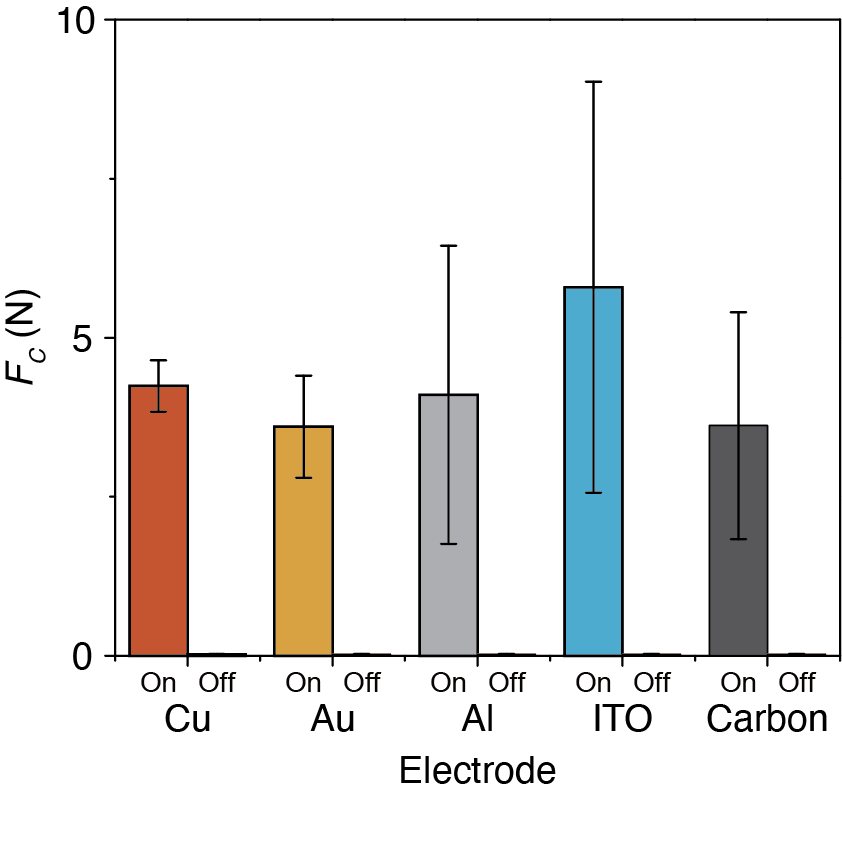
**

**Figure S11. Electrochemical adhesion behavior of PFSA ionomer on different conductive substrates in the "on" state (6 mC/cm²) and "off" state (– 6 mC/cm²).** All tests were conducted at a loading speed of 0.02 mm/s, using an adhesion area of 1 cm × 1 cm with a free length of 3.5 cm. Each sample was tested three times under both the "on" state (6 mC/cm²) and "off" state (–6 mC/cm²) after charging by chronopotentiometry.

**
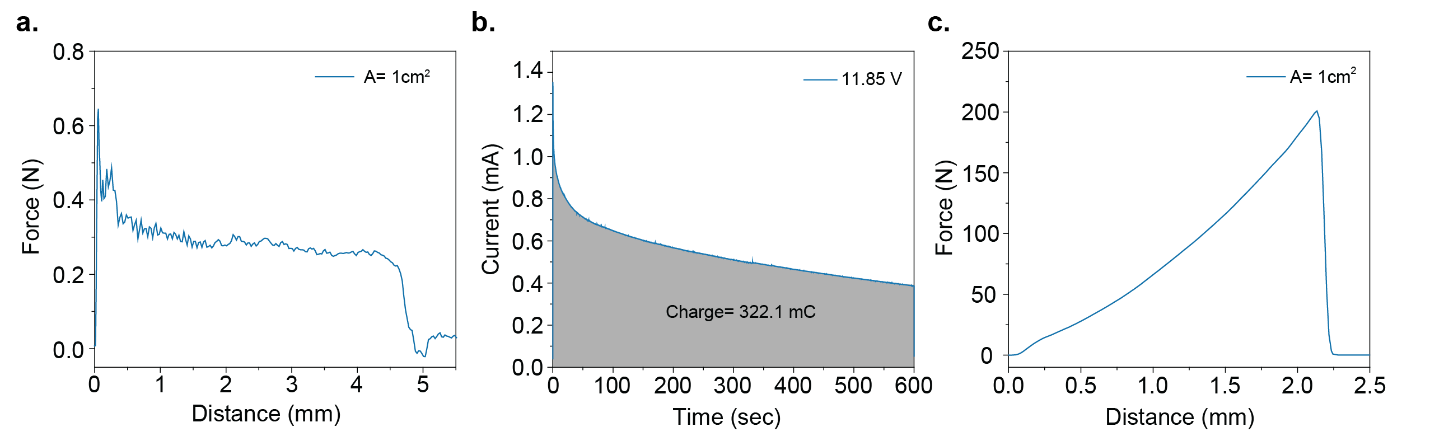
**

**Figure S12. Best performance result of PFSA ionomer electrochemical adhesive. a.** Tensile test under ‘off’ state test with *F_c_* per unit area of 0.435 N/cm^2^. **b.** Chronopotentiometry data of ‘on’ state. **c.** Tensile test under ‘on’ state test with *F_c_* per unit area of 200 N/cm^2^.

**Table S1.** Property of each material substance used for the *C_total_* calculation of the lap shear test

| **Material** | **Young’s Modulus (*E*)**  **[N/mm^2^]** | **Thickness (*t*)**  **[mm]** | **Poisson’s Ratio (𝞾)** |
| --- | --- | --- | --- |
| PFSA Ionomer | 130 | 0.146 | 0.34 |
| Copper | 130 x 10^3^ | 0.022 | 0.38 |
| PET backing | 263 x 10 | 0.132 | - |

**Table S2.** *G_c_* Fitting of the electrochemical adhesive lap shear test result

| **Fitting Result**  (*G_c_* = **Slope** x Current Density(*i)* x Charging time(*t*) + **Intercept**) | **Slope**  **[J/C]** | **Intercept**  **[J/m^2^]** | **Adjusted**  **R-Square** |
| --- | --- | --- | --- |
| Time (*t*) variant under constant current density (100 μA/cm^2^) **(Figure 2c.)** | 3.332 x 10^-2^  ± 7.622 x 10^-4^ | 0.121  ± 0.003 | 0.98 |
| Current variant under constant charging time (60 sec) **(Figure 2d.)** | 3.207 x 10^-2^  ± 4.749 x 10^-4^ | 0.109  ± 0.002 | 0.97 |

**Table S3.** R-Square of electrochemical adhesive lap shear test result under geometrical dependence

| **Data** | **R-Square**  **(3 mC/cm^2^)** | **R-Square**  **(6 mC/cm^2^)** | **R-Square**  **(12 mC/cm^2^)** |
| --- | --- | --- | --- |
| Width (*W*) dependence under constant charge density **(Figure 2e.)** | 0.82 | 0.92 | 0.96 |
| Area (*A*) dependence under constant charge density **(Figure 2f.)** | 0.95 | 0.99 | 0.94 |

**Table S4.** XPS surface analysis result of the PFSA ionomer electrochemical adhesive surface. Property of each material substance used for the *C_total_* calculation of the lap shear test.

| **Sample** | **Sulfonate Bond**  **(-SO_3_^-^)**  **[atomic concentration, %]** | **Cu - Sulfonate Bond**  **(Cu(-SO_3_^-^)_2_)**  **[atomic concentration, %]** | **Ether Bond**  **(C-O-C)**  **[atomic concentration, %]** |
| --- | --- | --- | --- |
| Bare  PFSA Ionomer | 57.62 | - | 42.38 |
| PFSA Ionomer  after Electrochemical Reaction  (10 min, current overload) | 1.33 | 59.92 | 38.95 |
| PFSA Ionomer  after Electrochemical Reaction  (30 sec, 100 μA) | 42.94 | 17.48 | 39.57 |
| PFSA Ionomer  after Electrochemical Reaction  (60 sec, 100 μA) | 39.95 | 19.32 | 41.63 |
| PFSA Ionomer  after Electrochemical Reaction  (120 sec, 100 μA) | 45.94 | 18.90 | 35.18 |
| PFSA Ionomer  after Electrochemical Reaction  (300 sec, 100 μA) | 34.97 | 23.77 | 41.26 |
